# A comparative study on the age, growth, and mortality of *Gobio huanghensis* (Luo, Le & Chen, 1977) in the Gansu and Ningxia sections of the upper Yellow River, China

**DOI:** 10.1186/s12862-024-02217-2

**Published:** 2024-03-06

**Authors:** Peilun Li, Jiacheng Liu, Yanbin Liu, Tai Wang, Kai Liu, Jilong Wang

**Affiliations:** 1 Heilongjiang River Fisheries Research Institute, Chinese Academy of Fishery Sciences, Harbin, 150070 China; 2 Scientific Observing and Experimental Station of Fishery Resources and Environment in Heilongjiang River Basin, Ministry of Agriculture and Rural Affairs, Harbin, 150070 China; 3 College of Animal Science and Technology, Northeast Agricultural University, Harbin, 150030 China; 4 Ningxia Fisheries Research Institute, Yinchuan, 750001 China; 5 Gansu Fisheries Research Institute, Lanzhou, 730030 China

**Keywords:** *G. Huanghensis*, Age, Growth, Mortality, Exploitation rate, Yellow River

## Abstract

**Balkground:**

*Gobio huanghensis* is a small economic fish endemic to the Yellow River at the junction of the Tibetan Plateau and the Huangtu Plateau in China. To understand the impact of environmental changes and human activities on the ecological structure of the *G. huanghensis* population, a comparative study was conducted on the age composition, growth characteristics, mortality rate, and exploitation rate of the *G. huanghensis* populations in the Gansu and Ningxia sections of the upper Yellow River.

**Results:**

During the investigation, a total of 1147 individuals were collected, with 427 individuals collected from the Gansu section and 720 individuals from the Ningxia section. The results showed that *G. huanghensis* in the Gansu section exhibited a total length ranging from 5.00 to 22.80 cm, with an average of 12.68 ± 4.03 cm. In the Ningxia section, the total length of *G. huanghensis* ranged from 2.15 to 20.65 cm, with an average of 9.48 ± 3.56 cm. The age composition of *G. huanghensis* in the Gansu section ranged from 1 to 7 years, where female fish were observed between 1 and 7 years old, and male fish between 1 and 6 years old. In the Ningxia section, both female and male fish ranged from 1 to 5 years old. The relationships between total length and body weight were (Gansu section, *R*^2^ = 0.9738) and (Ningxia section, *R*^2^ = 0.9686), indicating that fish in the Gansu section exhibit positive allometric growth, while fish in the Ningxia section exhibit negative allometric growth. The von Bertalanffy growth equation revealed that *G. huanghensis* in the Gansu section exhibited an asymptotic total length *L*_∞_ of 27.426 cm with a growth coefficient *K* of 0.225 yr^−1^, while in the Ningxia section, the asymptotic total length *L*_∞_ was 26.945 cm with a growth coefficient *K* of 0.263 yr^−1^. The total mortality rate (*Z*) values of *G. huanghensis* were 0.7592 yr and 1.1529 yr in the Gansu section and Ningxia section, respectively. The average natural mortality rate (*M*), estimated by three different methods, in the Gansu section was 0.4432 yr, while it was 0.5366 yr in the Ningxia section. The exploitation rate (*E*) of *G. huanghensis* was 0.4163 in the Gansu section and 0.5345 in the Ningxia section, indicating that the population in the Ningxia section may have been overexploited.

**Conclusion:**

Prolonged fishing pressures and environmental changes may have led to variations in the ecological parameters of the *G. huanghensis* population between the Gansu and Ningxia sections.

## Introduction

Habitat fragmentation is an important cause of species degradation and biodiversity loss [1–3]. An increasing number of species are forced to live in fragmented and shrinking habitats due to human expansion, resulting in changes in their population parameters, population size, and population viability [4, 5]. The construction of river water conservancy projects, especially cascade development, has damaged the continuity of rivers, resulting in fragmentation of aquatic habitats, hindering species migraten, dispersion, and communication, and causing a decline in biodiversity [3, 6]. Life history theory suggests that a population’s life history strategy is a result of balancing mature age, juvenile survival rate, and reproductive capacity [7]. Despite limitations imposed by genetic variation and evolutionary history, organisms’ energy allocation is balanced between growth, survival, and reproduction in response to different environmental conditions [8, 9]. Even within the same fish species, population characteristics may vary due to factors such as food availability, habitat characteristics, fishing pressure, and interspecific competition [10–12].

*G. huanghensis* is a small ecomnomic fish and belongs to the Cyprinidae, only distributed in the main and tributaries of the Yellow River at the junction of the Tibetan Plateau and the Huangtu Plateau in China [13]. *G. huanghensis* is primarily found in the shallows of river bays, where it predominantly consumes zoobenthos such as chironomid larvae and Gammarid. Additionally, it also includes phytoplankton and zooplankton in its diet [13]. As a result, this species holds significant importance in the water ecosystem, serving as a valuable food source for predatory fish. Since the 1960s, the population of *G. huanghensis* has experienced a sharp decline due to factors such as overfishing and the construction of hydropower stations [13]. These activities have led to the deterioration of the habitat environment and the shrinking of distribution waters for this species [14]. In 2016, *G. huanghensis* was listed as an endangered species in the “Red List of China’s Vertebrates” [15]. Although some studies have been conducted on *G. huanghensis*, these studies have primarily focused on resource investigation, geographical distribution, biological characteristics, ecological habits, and phylogeny [13, 14]. Unfortunately, investigations of the natural resources of the *G. huanghensis* population in the upper Yellow River have remained stagnant over the past three decades [13]. Essentially, human activities have gradually diminished the population of *G. huanghensis*. However, the lack of foundational biological studies has hindered the implementation of sound population management strategies.

The study of population characteristics such as age, growth, and mortality in fish is fundamental to fisheries resource assessment and management, as these characteristics are influenced by genetic factors and the natural environment [7, 16, 17]. Prolonged fishing pressures and environmental changes have resulted in variations in species diversity, community structure, and food webs in aquatic ecosystems and have influenced the dynamics of various hydrobios [18–20]. The potential changes in phenotypic characteristics and biological indicators of the *G. huanghensis* population under long-term survival pressure warrant our attention. To address these concerns, we conducted an investigation on the population resources of *G. huanghensis* in the upper reaches of the Yellow River. We selected the Gansu section and Ningxia section as research areas to investigate the population characteristics of *G. huanghensis*, including age structure, growth characteristics, mortality rate, and exploitation rate. Moreover, we aimed to explore the variations in these population characteristics among different habitats. Ultimately, this research provides a research foundation for the scientific management and conservation of resources.

## Materials and methods

### Investigation area

**Fig. 1:**
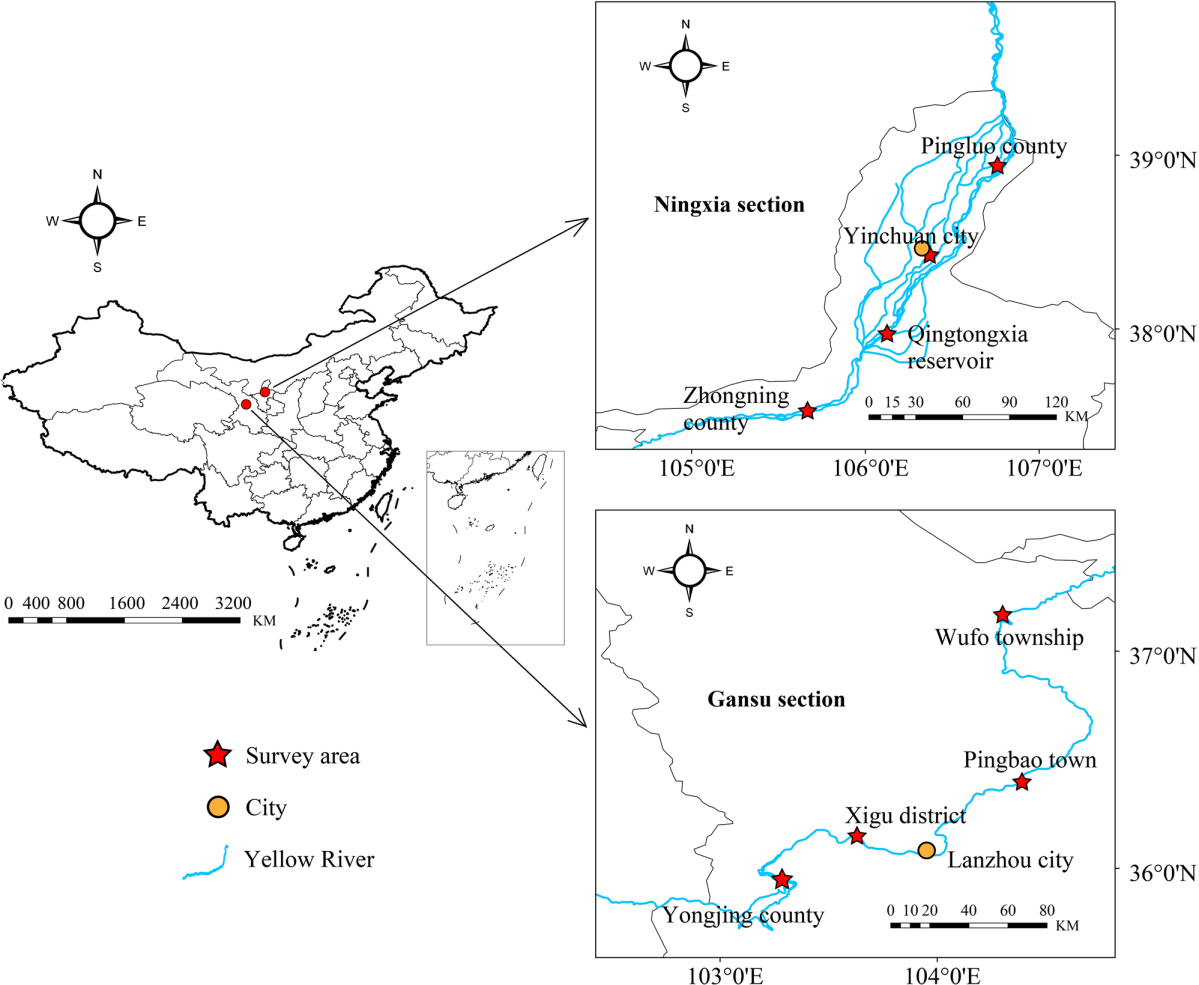
Sampling locations of *G. huanghensis* in the upper reaches of the Yellow River

The Yellow River, the second-largest river in China, originates from the Bayan Har Mountains on the Qinghai-Tibet Plateau. The main stream has a total length of 5464 km, and the basin area spans approximately 796,000 square kilometers. The upper reaches of the Yellow River can be divided into three sections based on the characteristics of the river channel: the source area, the gorge section, and the alluvial plain area. Among them, the gorge section is mainly located within Gansu Province, which is known as a concentrated distribution area of hydropower stations [21], while the area within Ningxia Hui Autonomous Region is primarily an alluvial plain area. This study was conducted in the upper reaches of the Yellow River, which spans from Yongjing county in Gansu Province to Pingluo county in the Ningxia Hui Autonomous Region (Fig. 1).

### Sampling and processing

Between July-October 2022, February-March 2023, and May 2023, a total of 1147 *G. huanghensis* specimens were collected from the investigation area. The collection techniques included the employment of gillnets with mesh sizes ranging from 1 to 4 cm and cage nets with dimensions of 15 cm in length, 40 cm in width, and 40 cm in height. The fish samples, taking from the fishing nets in their natural environment, suffered rapid mortality due to oxygen and water deprivation shortly after removal (no anesthetic was used). Following their demise, the total length (*L*) and body weight (*W*) of the samples were measured with accuracies of 0.01 cm and 0.01 g, respectively. Biological anatomy was assessed to distinguish between males and females based on gonadal morphology. The sagitta otoliths were extracted from the fish’s inner ear sac using tweezers. After removing the surface connective tissue, the otoliths were preserved in a centrifuge tube filled with a 95% ethanol solution and numbered accordingly. Water temperature was measured throughout the survey using a portable water quality tester (HACH, Loveland, CO, USA).

### Age estimation

First, the otoliths were coated with transparent nail polish and polished using sandpaper with a grit size of 1500–2000 until the growth center became clearly visible. Throughout the polishing process, the otoliths were examined under an optical microscope. Subsequently, the otolith sections were rinsed with anhydrous ethanol, rendered transparent with xylene, and sealed with neutral gum. Finally, the annual rings were observed and enumerated under an optical microscope to determine the characteristics of each ring. Furthermore, the age of each otolith was determined through a blind examination, employing the method described by Wang et al. [22].

### Length-weight relationships

The power function model, $$ {W}{ = }{a}{{ }{L}}^{{b}}$$, was used to establish the relationship between body weight (*W*) and total length (*L*). In this model, parameter *a* represents the growth condition factor, while parameter *b* represents the allometric growth factor. A *t*-test was performed at a significance level of 0.05 to evaluate the slope (*b*) of the length-weight relationship and determine if the obtained value significantly deviated from the value “3” [23].

### Growth characteristics

The von Bertalanffy growth equation was used to model the growth characteristics of *G. huanghensis*. The length growth formula is.

${L_t}={L_\infty }\left[ {1 - {e^{ - K(t - {t_0})}}} \right]$, where *L*_t_ denotes the total length of the fish at age *t* (cm), *L*_∞_ represents the asymptotic total length (cm), *K* denotes the growth coefficient (yr^−1^), *t* stands for the age of the fish sample (yr), and *t*_0_ is the theoretical initial age when the total length is zero (yr). The formula *φ*=lg*K*+2lg*L*_∞_ was used to calculate the growth characteristic index (*φ*), where *K* and *L*_∞_ are parameters from the von Bertalanffy growth equation. Moreover, the residual sum of squares (ARSS) was employed to statistically compare the fitted growth curves between sexes [24].

### Mortality and exploitation rate

An age-based catch curve analysis was used [25] to estimate the total mortality rate (*Z*). Catch curves were created by plotting the natural logarithm of the number of sampled fish in each age class against their respective age class. Only age classes that were fully recruited to the fishing gear were considered for *Z* estimation. The estimation of *Z* involved fitting a linear regression equation “$$ {y = mx }{+}{ }{n}$$” to the right limb of the catch curve, and the absolute value of the slope (*m*) in this equation represents the value of *Z* [26].

The natural mortality rate (*M*) was estimated using three different approaches. First, the length-based empirical relationship proposed by Pauly [27] is In *M*=−0.0066−0.279 In *K*+04634 In T, where *T* denotes the annual habitat temperature (℃) of the water where the fish stocks reside. *L*_∞_ and *K* are the asymptotic length and average curvature of the von Bertalanffy growth equation, respectively. Second, the age-based method proposed by Ralston [28] employs a regression approach and is $$ {M}{ = 0.0189 + 2.06}{K}$$, where *K* represents the average curvature of the von Bertalanffy growth equation. Last, the age-based method introduced by Zhan [29] is $$ {M}{ = -0.0021 + 2.5912/}{{t}}_{{m}}$$, where *t*_*m*_ indicates the observed maximum age in years.

The fishing mortality rate (*F*) was calculated by subtracting the natural mortality rate (*M*) from the total mortality coefficient (*Z*). Mathematically, this is expressed as $$ {F}{ = }{Z}{ - }{M}$$. Additionally, the population exploitation rate (*E*) was determined by dividing the fishing mortality rate (*F*) by the total mortality rate (*Z*): $$ {E}{ = }{F}{ / }{Z}$$. These formulas provide a method to evaluate the impact of fishing on the population by comparing fishing mortality to the overall mortality rate. The exploitation rate (*E*) represents the proportion of the total mortality attributed to fishing operations [30].

### Statistical analyses

The statistical analyses were performed using both Microsoft Excel 2016 and SPSS Statistics 19.0. Graphs were generated using Microsoft Excel 2016 and GraphPad Prism 8.0.

## Results

### Population structure

**Fig. 2:**
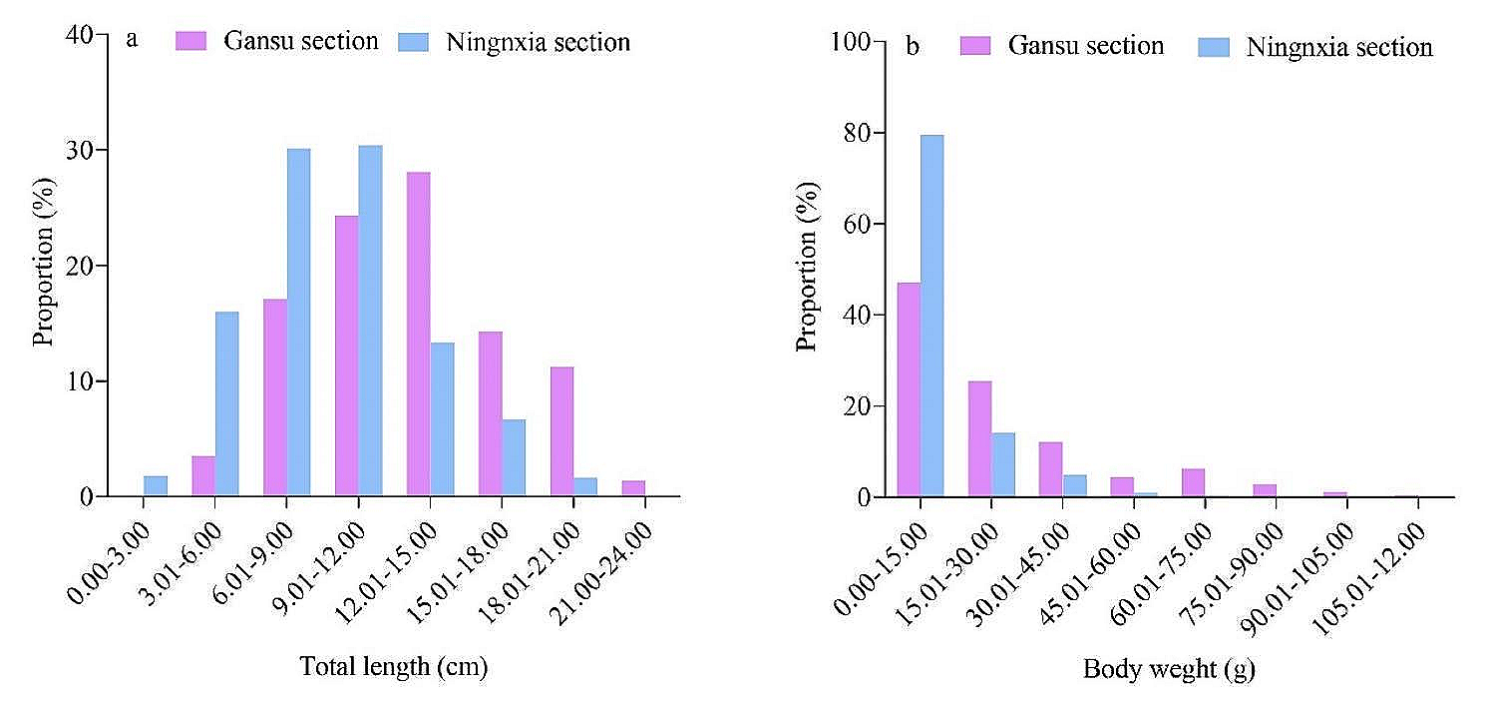
The distribution of total length (a) and body weight (b) of *G. huanghensis*

During the investigation, a total of 1147 samples were collected. Among them, there were 427 samples from the Gansu section, consisting of 149 females, 151 males, and 127 samples of unknown sex. From the Ningxia section, there were 720 samples, including 205 females, 203 males, and 321 samples of unknown sex. The *G. huanghensis* in the Gansu section exhibited a total length ranging from 5.00 to 22.80 cm, with an average of 12.68 ± 4.03 cm. In the Ningxia section, the total length of the *G. huanghensis* ranged from 2.15 to 20.65 cm, with an average of 9.48 ± 3.56 cm (Fig. 2a). The Mann-Whitney U test results showed a significant difference (*Z* = − 12.565, *P* < 0.01) in the total length of *G. huanghensis* between the Gansu and Ningxia sections. In terms of weight, the *G. huanghensis* in the Gansu section had a weight range of 0.96–117.42 g, with an average of 23.63 ± 22.50 g. On the other hand, in the Ningxia section, their weight ranged from 0.18 to 63.01 g, with an average of 10.66 ± 10.38 g (Fig. 2b). Mann-Whitney U test revealed a significant difference (*Z* = − 11.263, *P* < 0.01) in body weight between the Gansu and Ningxia sections. Regarding the length distribution, in the Gansu section, the majority (69.56%) of the total length of the *G. huanghensis* fell within the range of 3.01 to 15.00 cm. Similarly, in the Ningxia section, the majority (76.53%) of the total length was concentrated between 3.01 and 12.00 cm. In both the Gansu and Ningxia sections, the weight of *G. huanghensis* was mainly below 30.00 g. Specifically, the Gansu section accounted for 72.60% of the total samples, while the Ningxia section accounted for 93.61%.

### Age structure

**Fig. 3:**
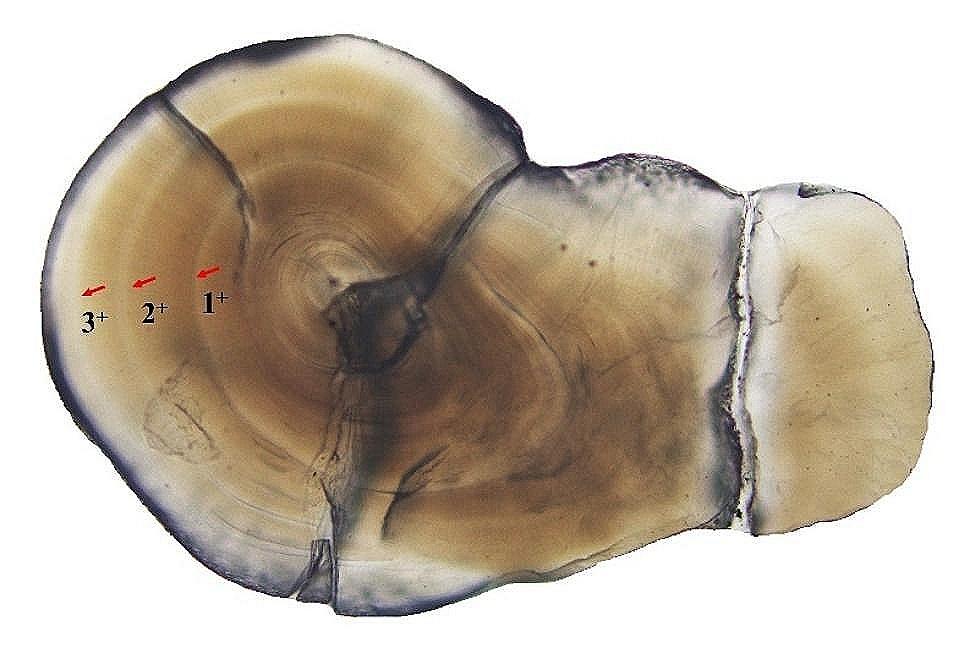
Otolith section of *G. huanghensis* (from Ningxia section, female, with a total length of 17.21 cm, body weight of 44.10 g, and 4 years old) in the upper reaches of the Yellow River. Note: the red arrows indicate that the otolith ring pattern consists of three light and dark rings, so the age of the sample is 3^+^ years old (4 years old)

**Fig. 4:**
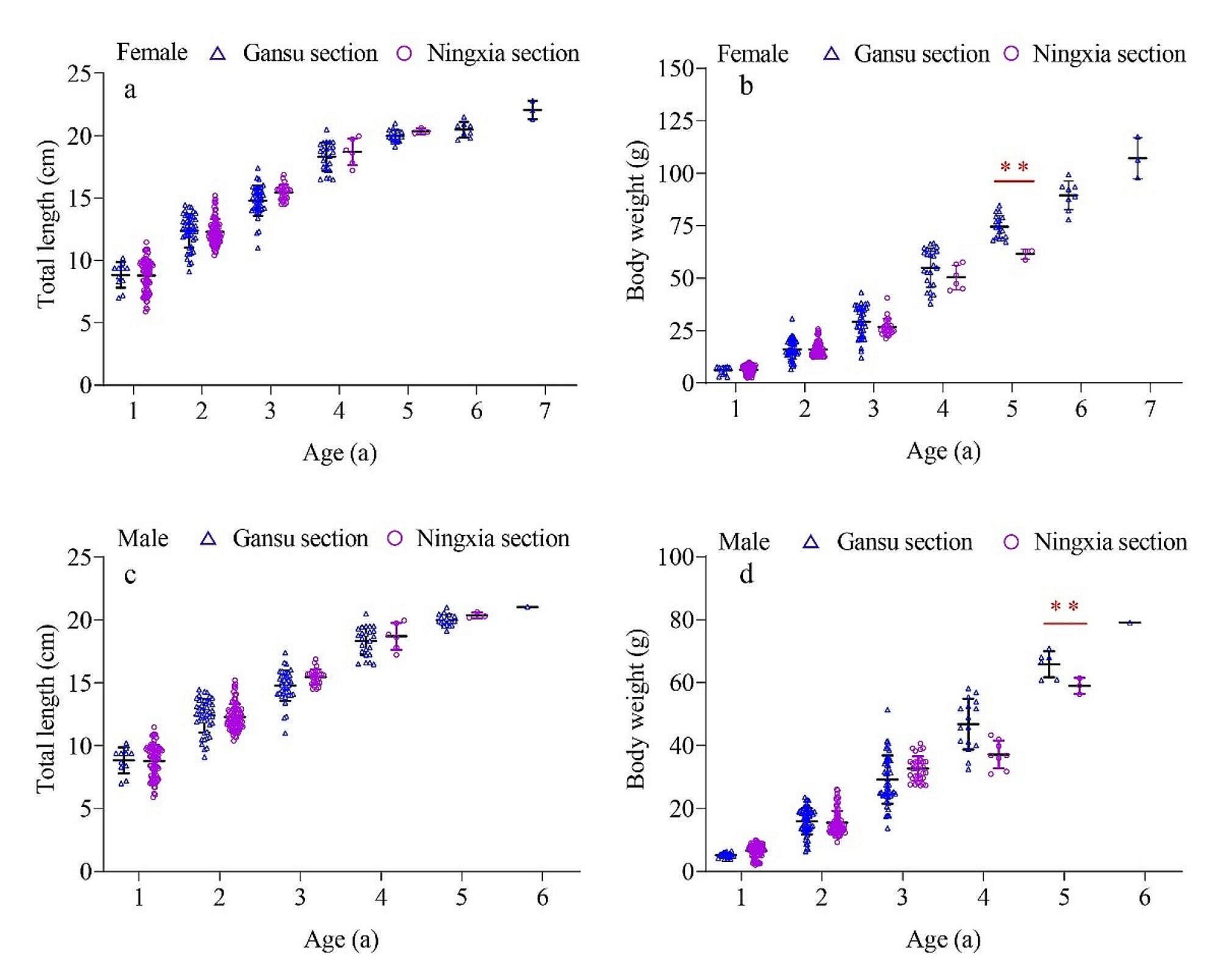
Distribution of total length and body weight of *G. huanghensis* in different age groups in Gansu and Ningxia sections (a and b for females, c and d for males). *Note* *indicates a significant difference (*P* < 0.05)

Following the grinding process of the otolith from *G. huanghensis*, a distinct ring pattern consisting of alternating dark and bright areas becomes visible when observed under a microscope. Specifically, the presence of a wide dark zone adjacent to a narrow bright zone indicated the formation of a growth ring (Fig. 3). The age composition of *G. huanghensis* in the Gansu section ranged from 1 to 7 years, where female fish were observed between 1 and 7 years old, and male fish between 1 and 6 years old. In the Ningxia section, both female and male fish ranged from 1 to 5 years old. Statistical analysis using Mann-Whitney U test revealed significant differences (*Z* = − 13.284, *P* < 0.01) in the age distribution of *G. huanghensis* between the Gansu and Ningxia sections. According to Table 1, it was evident that the average total length of the fish samples (ages 3, 4, and 5) from the Ningxia section was higher than that of the samples from the Gansu section. However, the average weight of the fish in the Ningxia section was relatively lower than that in the Gansu section, and the 5-year-old fish in the Gansu section showed a significantly greater weight than those in the Ningxia section (Fig. 4).

**Table 1:** Numbers of samples and total length (*L*) and body weight (*W*) in different ages of *G. huanghensis* in Gansu and Ningxia sections

Sexual	Gansu section				Ningxia section			
	Age (yr)	Samples (*n*)	*L* (cm)	*W* (g)	Age (yr)	Samples (*n*)	*L* (cm)	*W* (g)
Female	1	11	8.84 ± 1.03	6.04 ± 1.95	1	83	8.79 ± 1.27	6.34 ± 1.85
	2	45	12.38 ± 1.34	15.99 ± 4.78	2	87	12.28 ± 1.04	15.96 ± 3.25
	3	41	14.78 ± 1.21	29.21 ± 7.40	3	26	15.46 ± 0.61	26.61 ± 3.99
	4	24	18.32 ± 1.14	54.92 ± 9.26	4	6	18.69 ± 1.06	50.34 ± 5.83
	5	17	19.98 ± 0.47	74.49 ± 5.50	5	3	20.36 ± 0.23	61.49 ± 2.45
	6	8	20.47 ± 0.62	89.44 ± 6.89				
	7	3	22.05 ± 0.74	107.22 ± 9.81				
Male	1	19	8.70 ± 0.54	5.12 ± 0.82	1	86	8.75 ± 1.27	6.61 ± 2.07
	2	61	12.33 ± 1.14	15.88 ± 4.19	2	75	11.79 ± 1.39	15.44 ± 3.79
	3	48	14.82 ± 1.26	29.16 ± 7.67	3	31	15.23 ± 0.75	32.65 ± 3.89
	4	16	17.22 ± 1.28	46.82 ± 8.03	4	8	17.53 ± 0.58	37.17 ± 4.43
	5	6	19.82 ± 0.64	65.85 ± 4.12	5	3	20.10 ± 0.37	58.94 ± 2.54
	6	1	21.02 ± 0.00	79.10 ± 0.00				
Unknown	1	95	7.84 ± 1.57	4.38 ± 2.28	1	303	6.74 ± 2.18	4.19 ± 2.81
	2	32	11.13 ± 0.90	11.90 ± 2.52	2	9	10.99 ± 0.48	10.89 ± 0.48

### Length-weight relationship

**Fig. 5:**
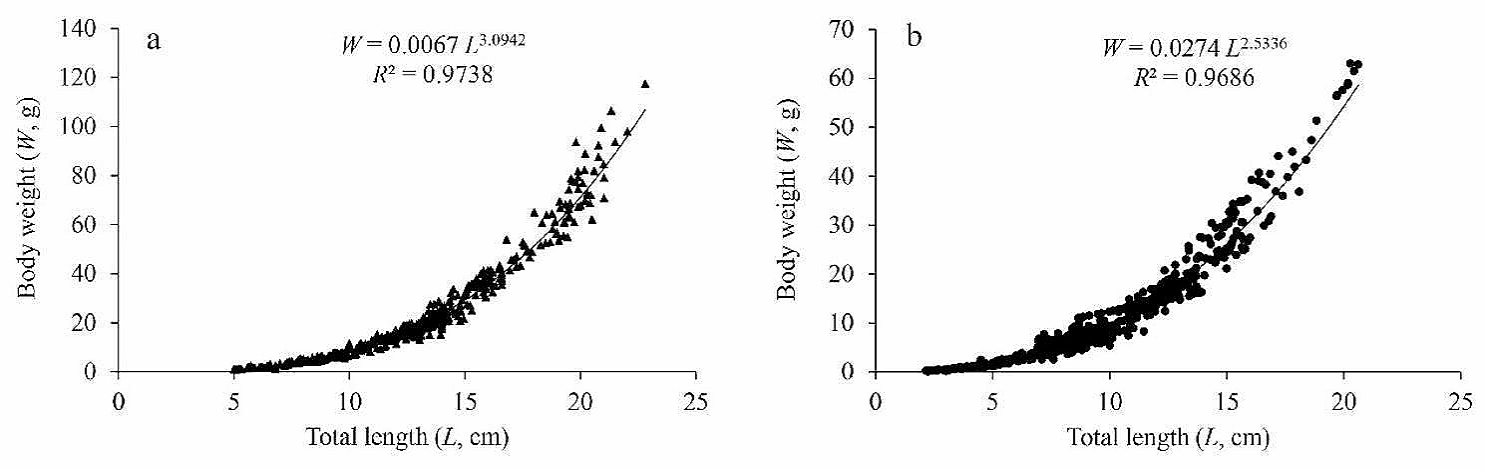
Length-weight relationship of *G. huanghensis* in the Gansu section (a) and Ningxia section (b)

The association between the total length and body weight of *G. huanghensis* was fitted using the power function equation, and the samples from the Gansu section and Ningxia section were fitted separately. The formula was as follows: $$ {W}{ = 0.0067}{{ L}}^{{3.0942}}$$ (Gansu section, *R*^2^ = 0.9738) (Fig. 5a), $$ {W}{ = 0.0274 }{{L}}^{{2.5336}}$$ (Ningxia section, *R*^2^ = 0.9686) (Fig. 5b). The *b* value in the Gansu section was significantly higher than “3” (*t*-test, *t* = 2.1497, *P* < 0.05), while in the Ningxia section, it was significantly lower than “3” (*t*-test, *t* = 24.2127, *P* < 0.05). These findings indicate that the fish population in the Gansu section demonstrates a positive allometric growth pattern, whereas the fish population in the Ningxia section displays a negative allometric growth pattern.

### Growth equation

**Fig. 6:**
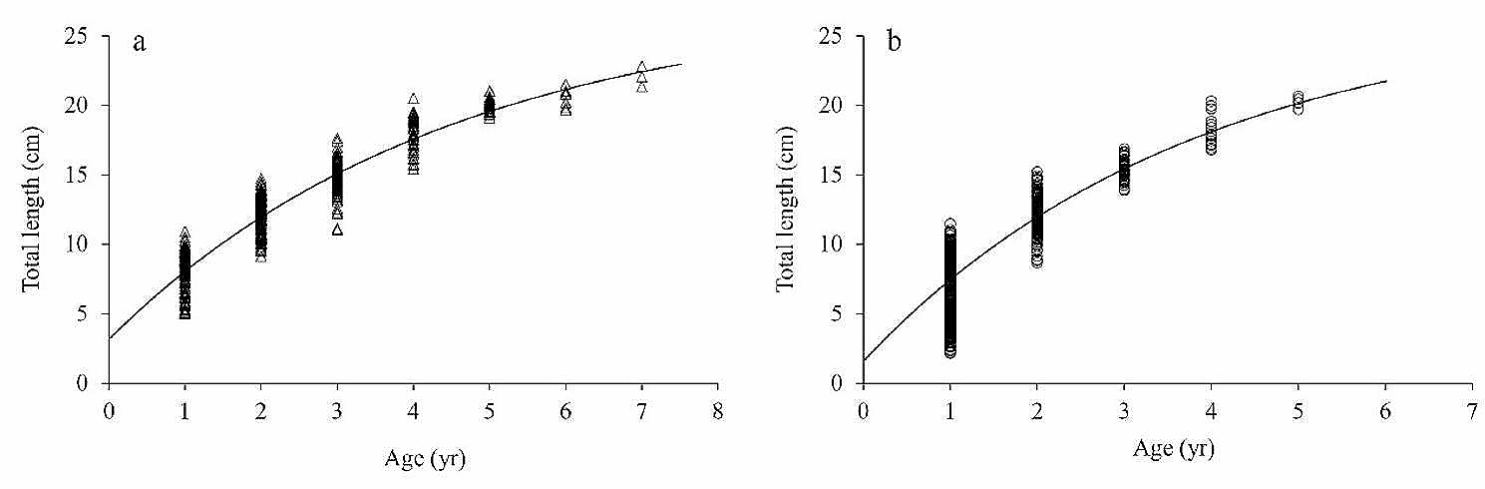
Von Bertalanffy growth curve fitted to total length-at-age for *G. huanghensis* from samples captured in the Gansu section (a) and Ningxia section (b)

The ARSS test was performed to assess the growth disparities between male and female *G. huanghensis* in the Gansu section (ARSS test: *F* = 0.0064, *P* = 1.0064) and Ningxia section (ARSS test: *F* = 0.0151, *P* = 1.0152). The results indicated no significant difference in growth between the sexes. Hence, the length growth equation for the entire sample set in the Gansu section could be represented as: ${{L}}_{{t}}{ = 27.426 }\left[{1-}{{e}}^{{-0.225}\left({t}{ + 0.55}\right)}\right]$ (*R*^2^ = 0.897), while the length growth equation for the entire sample set in the Ningxia section could be written as: ${{L}}_{{t}}{ = 26.945 }\left[{1-}{{e}}^{{-0.263}\left({t}{ + 0.236}\right)}\right]$ (*R*^2^ = 0.730). The curve of the total length growth fitted by the VBGF is shown in Fig. 6. The growth characteristic index (*φ*) for *G. huanghensis* in the Gansu section was determined to be 2.2285, whereas in the Ningxia section, the *φ* value was calculated to be 2.2809.

### Mortality and exploitation rate

**Fig. 7:**
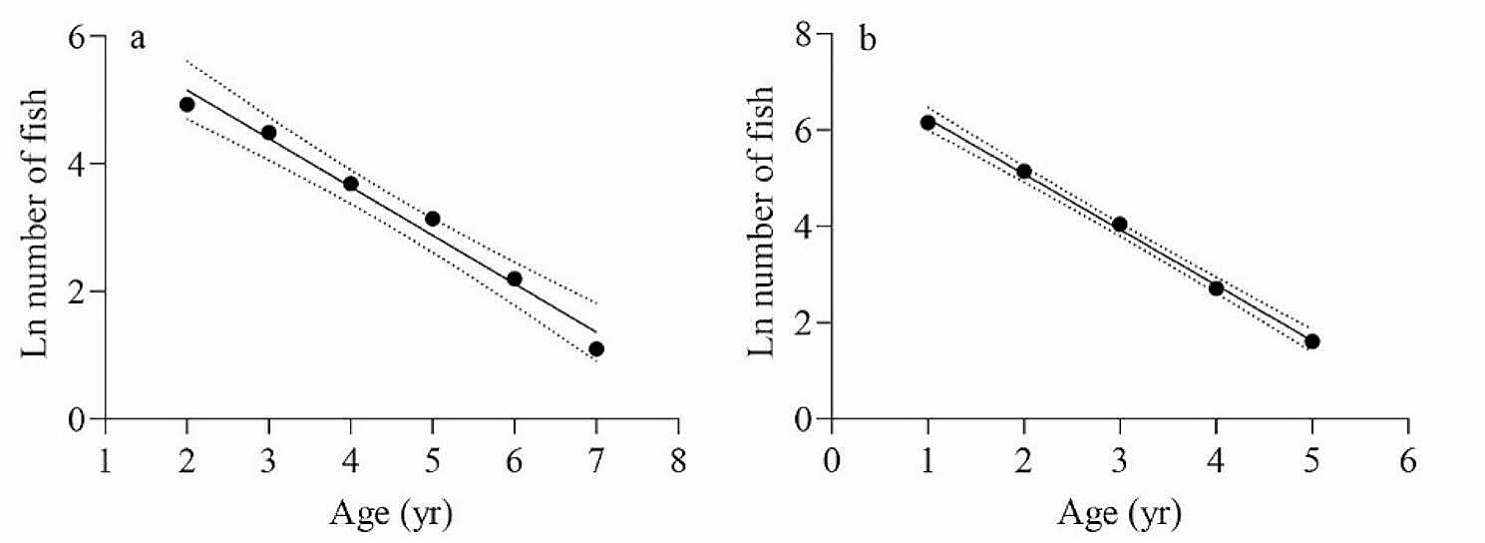
Catch curve based on observed age for G. huanghensis samples in the Gansu section (**a**) and Ningxia section (**b**). *Note* Z represents the total mortality rate

According to Pauly et al. [25], the collection of age 1 individuals during the survey in the Gansu section was limited. Consequently, when evaluating the total mortality rate (*Z*) of *G. huanghensis* in the Gansu section, data from the age 1 group were excluded. Conversely, in the Ningxia section, data from all age groups were fully captured, enabling the evaluation of *Z* across individuals of all ages in this section. Therefore, the *Z* values of *G. huanghensis* were 0.7592 yr and 1.1529 yr in the Gansu section and Ningxia section, respectively (Fig. 7). The average water temperature (*T*) in the investigation area was measured to be 12.5 °C. According to Pauly [27], Ralston [28], and Zhan et al. [29], the natural mortality rates (*M*) for the total samples in the Gansu section were 0.4790 yr, 0.4824 yr, and 0.3681 yr, respectively. Similarly, for the total samples in the Ningxia section, the natural mortality rates (*M*) were 0.5331 yr, 0.5607 yr, and 0.5161 yr, according to Pauly [27], Ralston [28], and Zhan et al. [29], respectively. Therefore, it was determined that the average *M* value in the Gansu section was 0.4432 yr, while it was 0.5366 yr in the Ningxia section. As a result, the fishing mortality (*F*) for the total samples in the Gansu section was computed as 0.3160 yr, with an exploitation rate (*E*) determined as 0.4163. Similarly, in the Ningxia section, the fishing mortality (*F*) for the total samples was calculated as 0.6163 year, with an exploitation rate (*E*) determined as 0.5366.

## Discussion

*G. huanghensis* is the only fish of the *Gobio* genus found in the upper reaches of the Yellow River [13]. Its meat is tender and delicious, making it an important small-scale economic fish in the region [14]. In our study, the maximum total length and body weight for females were 22.80 cm and 117.42 g, respectively, while for males, the maximum values were 21.02 cm and 79.10 g, respectively. Females of this species generally had a larger body size, with an average total length of 13.12 ± 3.76 cm, whereas males had an average total length of 12.29 ± 3.19 cm. The phenomenon of sexual dimorphism, with females being larger than males, has been documented in various teleosts, such as *Gymnocypris firmispinatus* [3], *Lateolabrax latus* [16], and *Coregonus ussuriensis* [22]. Fish age estimation is fundamental in the study of fish biology and ecology [31]. It serves as a crucial foundation for analyzing population dynamics, evaluating changing trends, and understanding fish growth, age distribution, and sexual maturity. Currently, otoliths are the most widely and accurately used material for age identification in fish [3, 22, 32]. In this study, we used otoliths to estimate the age of *G. huanghensis*, revealing that the age composition of *G. huanghensis* in the upper reaches of the Yellow River ranged from 1 to 7 years old. Among them, ages 1 and 2 were the main age groups, accounting for approximately 78.99% of the total samples and displaying characteristics associated with younger age. During our investigation, we found that the age range of *G. huanghensis* in the Gansu section ranged from 1 to 7 years old, whereas in the Ningxia section it ranged from 1 to 5 years old. Additionally, the age distribution in these two sections differed significantly from one another. Zhan [33] indicated that population replenishment can partially offset the decline in population through proper fishing practices. Nevertheless, overfishing disturbs the balance of the population, resulting in a substantial decrease in population and the depletion of resources, particularly affecting older fish. Compared to the Gansu section, the Ningxia section of the Yellow River showed a significant decrease in the number of older *G. huanghensis*, with a higher proportion of younger *G. huanghensis*. Therefore, our conclusion implies that the number of large individuals of fish is lower in the Ningxia section compared to the Gansu section, possibly due to excessive fishing. Moreover, this phenomenon may potentially be influenced by additional variables such as the environment feature, food abundance, and sample variances.

Fish growth refers to the accumulation of materials and energy within fish, resulting in changes in their length and weight. Different growth patterns give rise to distinct characteristics in fish growth, and the diverse habitats they inhabit contribute to their varying ecological adaptability [34, 35]. According to Li et al. [36], different environmental conditions in different bodies of water might affect the growth parameters of fish populations. The availability of food organisms, water temperature, fishing pressure, and interspecific competition pressure are a few of these variables. As a result, these variances may cause various fish populations to exhibit distinctive growth patterns. The *b* value in the relationship between length and weight indicates the growth characteristics of fish in various stages of development and different environmental conditions [37]. It was reported that valid *b* values should range from 2.50 to 3.50 [38]. Therefore, the provided *b* values for several Gobioninae fishes ranged from 2.53 to 3.20, indicating their validity (Table 2). In this study, the *b* value of *G. huanghensis* in the Gansu section was 3.0942, indicating a positive allometric growth pattern, similar to *Rhinogobio cylindricus* [39], *R. ventralis* [2], and *Coreius heterodon* [40]. However, the *b* value of *G. huanghensis* in the Ningxia section was 2.5336, indicating a negative allometric growth pattern, similar to *Saurogobio dabryi* [41] and the female *Pseudorasbora parva* [42]. According to Gisbert [43], allometric growth refers to the uneven development of different functional organs in fish as a result of their adaptation to the external environment. Lu et al. [44] suggest that the current increase in fishing intensity and the use of smaller mesh size in fishing nets have led to a shift in target species toward small and medium-sized fish, which are then processed into fishmeal for profit. For *G. huanghensis*, the Ningxia section exhibited greater growth in total length relative to body weight while the Gansu section exhibited superior growth in body weight relative to total length. This change may be attributed to the increase in fishing intensity and the use of smaller mesh sizes in fishing nets. So, in the Ningxia section, fishing nets may be selectively permitting slender-bodied individuals to survive, as they are better equipped to evade the nets.

Furthermore, the *K* values of *G. huanghensis* in the Gansu section and Ningxia section were found to be 0.225 yr^− 1^ and 0.236 yr^− 1^, respectively. It is evident from Fig. 2 that these values are lower than those of other Gobioninae fishes such as *S. dabryi* [41], *R. ventralis* [2] and *Squalidus argentatus* [47]. On the other hand, these values are higher than those reported for *R. cylindricus* [39] and *C. heterodon* [40]. Campana [45] indicated that the extreme values of the sample length are the primary factor affecting the *K* value. Consequently, the Gobioninae fishes exhibit diversity in their *K* values, which is influenced not only by environmental factors but also by the size of individual samples. Munro and Pauly [46] indicated that the values of *L*_∞_ and *K* are combined to create the growth characteristic index (*φ*), which has a positive correlation with growth rate and may be used to compare the growth performance of fish belonging to the same subfamily. The *φ* values in our study were 2.2285 and 2.2809 in the Gandu section and Ningxia section respectively, higher than those of *P. parva* [42] and *S. argentatus* [47], lower than *S. dabryi* [41], *R. ventralis* [2], *C. heterodon* [40] and *R. cylindricus* [39]. In general, *G. huanghensis*, a species within the Gobioninae family, exhibits slow growth characteristics in the upper reaches of the Yellow River. These slow growth characteristics may be attributed to factors such as low water temperature, high sediment content in the water body, and a limited abundance of food organisms in the investigated area [7, 9].

**Table 2:** Growth parameters among several Gobioninae fishes in China

Species	Investigation area	Sexual	Growth parameters				Source
			b	L_∞_ (cm)	K (yr^− 1^)	φ	
*Saurogobio dabryi*	Angu Reservoir of Dadu River	♀&♂	2.8700	24.200	0.510	2.4752	[41]
*Rhinogobio cylindricus*	Upper Yangtze River	♀&♂	3.0990	34.878	0.180	2.3404	[39]
*Rhinogobio ventralis*	Upper Yangtze River	♀&♂	3.2000	33.800	0.240	2.4380	[2]
*Coreius heterodon*	Middle Yangtze River	♀&♂	3.1100	48.270	0.210	2.6896	[40]
*Pseudorasbora parva*	Upper Huaihe River	♀	2.9280	10.701	0.246	1.4497	[42]
		♂	3.1160	14.525	0.181	1.5819	
*Squalidus argentatus*	Wuhu section of Yangtze River	♀	3.0600	14.831	0.250	1.7403	[47]
		♂	3.1690	12.136	0.350	1.7122	
*Gobio huanghensis*	Gansu section of Yellow River	♀&♂	3.0942	27.426	0.225	2.2285	This study
	Ningxia section of Yellow River	♀&♂	2.5336	26.945	0.236	2.2809	

Research on fish population dynamics has focused on various aspects such as population replenishment, mortality, resource assessment, and management strategies [48]. The fluctuations in fish population numbers are influenced by both environmental conditions and factors such as reproductive potential, growth rate, and mortality within the population itself [31]. Mortality, as a fundamental parameter, plays a pivotal role in driving changes in population dynamics by influencing the population’s overall quantity. According to Then et al. [49], various methods have been reported in the last 70 years to evaluate the natural mortality rate (*M*) of a stock, utilizing empirical evidence from comparative life history information. In this study, we used three methods to assess the value of *M*, which included the length-based empirical relationship proposed by Pauly [27], as well as the age-based methods proposed by Zhan et al. [29] and Ralston [28]. Thus, we concluded that the average *M* value in the Gansu section was 0.4432 yr, while it was 0.5366 yr in the Ningxia section. Beverton et al. [26] proposed that the *M*/*K* rate typically falls between 1.5 and 2.5, a widely acknowledged reasonable range. In this study, the *M*/*K* values for the Gansu section and Ningxia section were found to be 1.97 and 2.04, respectively, indicating that they fall within a reasonable range. Numerous factors impact fluctuations in fish population numbers, and fishing is one of the main causes. According to Zhan [33], population mortality in fish stocks is mainly influenced by natural causes when the *Z*/*K* rate is equal to or less than 3. However, when the *Z*/*K* rate exceeds 3, fishing activities become the primary contributor to population mortality. From our results, it appears that the values of *Z*/*K* in the Gansu section and Ningxia section were determined to be 3.37 and 4.38, respectively, both of which exceed 3. This suggests that the mortality of *G. huanghensis* in the upper reaches of the Yellow River is primarily attributed to fishing-related causes.

The exploitation rate (*E*) of fish is an important parameter in fishery management, and maintaining an exploitation rate of 0.5 is often considered a sustainable fishing practice according to Gulland [50]. According to the study, the exploitation rate of *G. huanghensis* was 0.4163 in the Gansu section and 0.5345 in the Ningxia section. These findings suggest that the population of *G. huanghensis* in the Ningxia section may have been overexploited under the current fishing intensity. During the investigation, it was observed that the Gansu section of the main stream of the Yellow River had a rugged terrain with steep mountains on both sides and a lack of shoals. This topography created difficulties for fishing operations, thereby playing an effective role in protecting fish resources. Differently, the Ningxia section exhibited relatively flat terrain on both sides of the river, with abundant shoals along the coast, making fishing more accessible. A variety of factors, including environmental conditions, human activity, behavioral patterns within the population, and climate fluctuations, can cause variances in fish populations [51]. Normally, in environments with relatively low fishing pressure, fish grow more slowly, and the average age and maximum age of individuals in the population increase. Conversely, environments subjected to higher fishing intensity experience significant declines in fish resources, particularly among older age groups. Consequently, these circumstances trigger alterations in population ecological parameters as ecological adaptations to substantial changes in the natural environment and intensified fishing practices undertaken by humans [31, 33, 52]. Therefore, we need to carry out more in-depth research to determine the status of resource exploitation of the *G. huanghensis* population in the upper reaches of the Yellow River, such as habitat characteristics, continuous population monitoring, and population genetic diversity.

## Conclusion

This study conducted a comparative analysis of the age, growth, and mortality rates of *G. huanghensis* in the Gansu and Ningxia sections of the upper Yellow River. It was found that *G. huanghensis* in the Gansu section ranged in age from 1 to 7, whereas it was only 1 to 5 years old in the Ningxia section. By evaluating the relationship between the total length and body weight of *G. huanghensis*, it was observed that the Gansu section exhibited positive allometric growth, whereas the Ningxia section displayed negative allometric growth. Additionally, our results also indicated that the population of *G. huanghensis* may have been overexploited in the Ningxia section. Prolonged fishing pressure and environmental changes may have led to variations in the ecological parameters of the *G. huanghensis* population between the Gansu and Ningxia sections. This study provides scientific data for ecological and conservation biology research on the *G. huanghensis* population in the upper reaches of the Yellow River.

## Data Availability

The data presented in this study are available on request from the corresponding author.
